# Diabetes and the kidney

**DOI:** 10.1016/j.clinme.2025.100512

**Published:** 2025-09-10

**Authors:** Sagen Zac-Varghese

**Affiliations:** Diabetes, Endocrinology and General Internal Medicine, East and North Herts Teaching NHS Trust, Lister Hospital, Coreys Mill Lane, Stevenage SG1 4AB, England

**Keywords:** Diabetes, Diabetic nephropathy, Diabetic kidney disease, Diabetes and chronic kidney disease

## Abstract

Diabetic kidney disease (DKD) management has become increasingly complex. For non-specialists in diabetes or kidney disease, there are some basic standards of care that apply to all people with DKD. These include blood glucose optimisation, blood pressure management, renin–angiotensin–aldosterone system inhibitors (RAASi), lipid management and smoking cessation. There are also risk prediction tools, such as the Kidney Failure Risk Equation, that can be used to guide management and referral to specialist care. This article provides a broad overview of DKD and signposts the reader to useful resources for people with DKD.

## Highlights

• Chronic kidney disease in people with diabetes is often a microvascular complication of diabetes.
• Consider an alternate diagnosis if there is good glycaemic control with no other complications, there is an active urine sediment, a rapid decline in eGFR or systemic manifestations of other conditions.
• Once diabetic kidney disease (DKD) is diagnosed, use the five-finger rule to enable people to have the standard pillars of care.
• Empower the person with DKD by providing access to relevant resources.
• Highlight the **sick day rules** for people with DKD and ensure that if medications are stopped due to intercurrent illness, there is a clear plan to restart these on recovery.

## Introduction

Chronic kidney disease (CKD) affects approximately one in three people with diabetes.^1^^,^^2^ The risk and severity of CKD varies according to the population studied, being more prevalent and more severe in areas of social deprivation, reflecting access to and quality of care.

### Definitions

CKD is defined as a sustained reduction in renal function, estimated glomerular filtration rate (eGFR) < 60 mL/min/1.73 m² or urine albumin-to-creatinine ratio (ACR) > 3 mg/mmol, for more than 3 months, or structural abnormalities of the renal tract.

In most cases, CKD is a microvascular complication of diabetes. It is recognised, however, that CKD within diabetes is heterogenous. It may be related to ageing, hypertension or obesity, for example. Because of this, nomenclature has changed. Instead of the term ‘diabetic nephropathy’, the terms ‘diabetic kidney disease’ (DKD) or ‘diabetes and CKD’ are favoured as umbrella terms to reflect the different aetiologies.

### Diagnosis of DKD

DKD is a clinical diagnosis and biopsy is unnecessary. However, people with diabetes might have CKD due to other pathologies. If glucose control is within target, there are no other diabetes-related complications, there is an active urine sediment, a rapid decline in eGFR or systemic manifestations of other conditions, an alternate diagnosis should be considered. A thorough history, medication review, examination and relevant investigations will help to exclude alternate diagnoses.

### Why does it matter?

The importance of early recognition and management of DKD is related to cardiovascular disease risk reduction. The often-quoted FinnDiane study highlights the correlation between the degree of kidney disease and the increased risk of cardiovascular disease and premature mortality. In type 1 diabetes, people with moderately increased albuminuria have a threefold increased risk of premature mortality, people with severely increased albuminuria have a ninefold increased risk and people with end-stage kidney disease (ESKD) have an 18-fold increased risk. A similar correlation is seen in people with type 2 diabetes. Importantly, regression of CKD reduces this risk.^3^

### A hidden issue

A significant number of people with DKD remain undiagnosed, untreated and unaware of their condition. Recognition of DKD and early treatment will reduce cardiovascular disease and improve mortality.

### Managing DKD

#### Monitoring renal function

The frequency of monitoring for people with CKD depends on the CKD stage (Table 1).^4^

**Table 1:** Frequency of monitoring in CKD (adapted from NICE).^4^

CKD stage		Urine ACR (mg/mmol)		
		A1 (< 3)	A2 (3–30) [Previously termed microalbuminuria]	A3 (> 30) [Previously termed macroalbuminuria]
eGFR (mL/min/1.73 m²)	G1–2 (>60)	≥12 months	12 months	≤ 12 months
	G3a (45–59)	12 months	12 months	6 months
	G3b (30–44)	6–12 months	6 months	≤ 6 months
	G4 (15–29)	6 months	6 months	4 months
	G5 (< 15)	3 months	3 months	≤ 3 months

Criteria for referral to a renal specialist have been proposed by NICE (Box 1).^4^ The Kidney Failure Risk Equation (KFRE) can be used to predict the risk of ESKD and the need for renal replacement therapy (Box 2).

**Box 1:** Criteria for referral to a renal specialist.

• Suspected rare renal or genetic conditions including vasculitis or autosomal dominant polycystic kidney disease. • Young age – higher lifetime risk of developing ESKD. • Rapidly declining eGFR or rising urine ACR (not caused by diabetes) or haematuria. • Complications of CKD eg anaemia, hypertension (requiring ≥ 4 antihypertensive medicines at therapeutic doses) or metabolic bone disease. • > 5% risk of needing renal replacement therapy in the next 5 years (Box 2, KFRE). • A sustained decrease in eGFR of ≥ 25% and a change in eGFR category within 12 months. • A sustained decrease in eGFR of ≥ 15 mL/min/1.73 m² or more per year. • Suspected renal artery stenosis.

**Box 2:** Useful resources for people with DKD.

**Type 1 diabetes** https://bertiediabetes.com/ **Type 2 diabetes** https://www.diabetes.org.uk/about-diabetes/looking-after-diabetes/education **General information on DKD** https://trenddiabetes.online/portfolio/diabetes-and-your-kidneys/ https://kidneyfailurerisk.co.uk/ **SGLT2i leaflet** https://kidneycareuk.org/get-support/free-resources/patient-information-booklets/sglt2-inhibitors/ https://guidelines.ukkidney.org/patient-information-leaflets/

**Box 3:** Example of sick day rules for people with type 2 diabetes.

**Type 2 diabetes sick day rules** **Diabetic medications to STOP when unwell.** **ACE inhibitors:** names ending in ‘pril’ - examples: lisinopril, perindopril, ramipril. **ARBs:** names ending in ‘sartan’ - examples: losartan, candesartan, valsartan. **Diuretics:** sometimes called ‘water pills’ - examples: furosemide, bendroflumethiazide, indapamide, spironolactone. **NSAIDs:** anti-inflammatory medications - examples: ibuprofen, naproxen, diclofenac. **Metformin** **SGLT2 inhibitors:** names ending in ‘flozin’ - examples: canagliflozin, dapagliflozin, empagliflozin, ertugliflozin. Restart your medication when you are well (normally after 24–48 hours of eating and drinking normally). **If you are on insulin, never stop insulin. Seek advice for dose adjustment.**

#### The five-finger rule

**Fig. 1:**
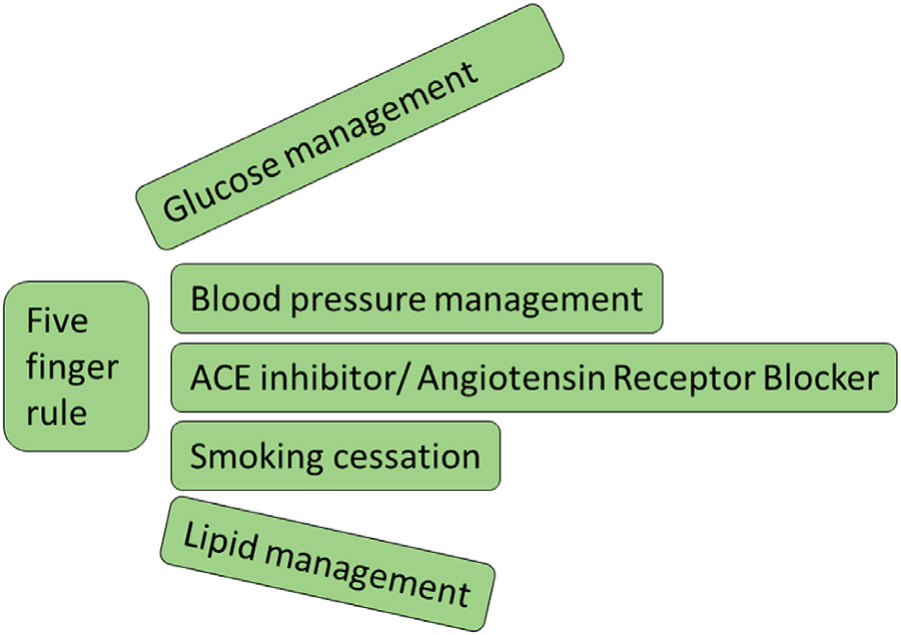
Five-finger rule: management of DKD.^5^

The five-finger rule is a useful aide memoire for all healthcare practitioners caring for people with DKD (Fig. 1).^5^

Small improvements in glucose, blood pressure (BP) and lipids lead to large improvements in morbidity and mortality.

The Steno-2 study, initiated in 1993, demonstrated that multifactorial intervention (including behavioural modification) led to a significant reduction in renal decline, cardiovascular disease and mortality. These benefits persisted beyond the duration of the study and were evident at 21 years of follow-up.^6^

##### Glucose management

Glucose management focuses on avoiding hyperglycaemia, avoiding hypoglycaemia and reducing glycaemic variability. Good glycaemic control has a positive effect on cardiorenal outcomes.

### Hypoglycaemia risk

People with DKD have a higher risk of hypoglycaemia due to reduced insulin metabolism and clearance. During fasting, 30% of gluconeogenesis comes from the kidney. Consequently, people with DKD are at higher risk of fasting hypoglycaemia.

People on dialysis have variable requirements for insulin. Dialysis improves uraemia and acidaemia, which reduces insulin resistance. To reduce the risk of hypoglycaemia, insulin doses can be reduced by 25% on dialysis days. A guideline on glucose management in dialysis is available from the Joint British Diabetes Society.^7^

### Anti-hyperglycaemic agents

In type 1 diabetes, insulin is currently the only available licensed medication. In type 2 diabetes, a number of medications are available, some with cardiorenal benefit.

#### Metformin

Metformin is safe, effective, carries no risk of hypoglycaemia and tends to be weight neutral. The dose needs to be halved if the eGFR is < 45 mL/min/1.73 m² and stopped when the eGFR is < 30 mL/min/1.73 m², due to the increased risk of lactic acidosis.

#### Sulfonylureas

Sulfonylureas accumulate in renal failure and increase the risk of hypoglycaemia. The dose should be minimised in renal failure to reduce this risk. They are highly protein bound, so unlikely to be dialysed and can cause post-dialysis hypoglycaemia.

#### Dipeptidyl peptidase-IV (DPPIV) inhibitors

DPPIV inhibitors can be used in CKD and are licensed for use in dialysis. These have no risk of hypoglycaemia and are weight neutral.

#### Glucagon-like peptide-1 receptor agonists (GLP-1 RA) and glucose-dependent insulinotropic polypeptide (GIP)/GLP-1 RA

GLP-1 and GIP are incretin hormones released from the gut that stimulate the release of insulin. Their effects are glucose dependent, so they do not cause hypoglycaemia when used alone.

There are currently five GLP-1 RA and one GIP/GLP-1 RA available in the UK. These have beneficial effects on glucose lowering and weight loss, and some (liraglutide, dulaglutide, semaglutide and tirzepatide) have additional cardiorenal benefits; a reduction in the risk of atherosclerotic cardiovascular disease and albuminuria.^8^, ^9^, ^10^, ^11^ Semaglutide has additional evidence for reduction in kidney failure, eGFR decline and death from cardiorenal disease.^8^

There are known side effects for GLP-1 RA and GIP/GLP-1 RA including gastrointestinal side effects and pancreatitis. There is also a risk of worsening retinopathy in people with active eye disease, known as early worsening of diabetic retinopathy (EWDR). This is notable for semaglutide and tirzepatide, and is likely due to the potential of these drugs to rapidly reduce HbA1c.

#### Sodium glucose cotransporter 2 inhibitors (SGLT2i)

SGLT2i exert cardiorenal protective effects through multiple mechanisms. Canagliflozin, dapagliflozin and empagliflozin are all beneficial in DKD, reducing the decline in eGFR, progression to ESKD and death from kidney failure by approximately 30–40%.^12^, ^13^, ^14^

SGLT2i can be started in people with an eGFR above 15–30 mL/min/1.73 m², depending on licensing, and continued until dialysis.

The risks of SGLT2i include urinary tract infections, mycotic genital infections and diabetic ketoacidosis. People starting SGLT2i should be counselled appropriately with regard to sick day rules (Box 2 and Box 3).

##### Blood pressure management

After diabetes, hypertension is the second largest cause of CKD and ESKD. A combination of diabetes and hypertension leads to a more rapid decline in kidney function and increased severity of disease.

The target for BP control is < 140/90 mmHg and < 130/80 mmHg for people with a higher risk, those with CKD and diabetes or a urine ACR > 70 mg/mmol.

For people without albuminuria, NICE guidelines for hypertension can be followed.

For those with albuminuria (A2 or A3), including those without hypertension, renin–angiotensin–aldosterone system inhibitors (RAASi) are favoured.^15^

##### Renin–angiotensin–aldosterone system inhibitors (RAASi)

In diabetes, increased glucose activates the RAAS, leading to the expression of proinflammatory and profibrotic mediators and glomerular hyperfiltration.

Angiotensin-converting enzyme inhibitors (ACEi) and angiotensin receptor blockers (ARB) reduce intraglomerular pressure, inflammation and fibrosis, and have benefits beyond BP reduction.

After starting or increasing the dose of an ACEi or ARB, urea and electrolytes should be checked within 2–4 weeks. An increase in creatinine can be predicted and RAASi can be continued unless the rise in creatinine is > 30%. If this occurs, evaluation for renal artery stenosis or volume depletion should take place.

It is important to highlight the **sick day rules** for people with DKD. If RAASi is stopped due to intercurrent illness, there should be a clear plan to restart this on recovery (Box 2 and Box 3).

Traditionally, RAASi have been stopped at lower eGFRs. The STOP ACEi trial demonstrated that continuing RAASi at lower eGFRs (< 30 mL/min/1.73 m²) does not affect the progression of renal failure.^16^

#### Finerenone

It is known that people on long-term RAASi can have ‘aldosterone escape’, where the aldosterone levels return to an elevated level.

Spironolactone, a classic aldosterone antagonist, is associated with side effects including hyperkalaemia, which are more prevalent in CKD. The BARACK-D-RCT found that 25 mg of spironolactone offered no cardiorenal protection, but was associated with an increase in adverse events.^17^

Finerenone is a third-generation mineralocorticoid receptor inverse agonist found to reduce cardiovascular disease and renal disease in people with DKD.^18^ It has reduced side effects compared to spironolactone.

Currently, finerenone is licensed for people with type 2 diabetes, with an eGFR between 25 and 60 mL/min, albuminuria (urine ACR > 3 mg/mmol), normokalaemia (K^+^ ≤ 5 mmol/L) and on maximally tolerated RAASi and SGLT2i. Finerenone should be stopped when the eGFR drops below 15 mL/min.

##### Lipid management

Large meta-analyses from the Cholesterol Treatment Trialists’ (CTT) Collaboration database demonstrated that statins reduce the risk of a first major vascular event by 21% per mmol/L reduction in LDL cholesterol.^19^ As eGFR declines, statin therapy becomes less effective, and smaller reductions in cardiovascular risk are seen.

The ABCD-UKKA have guidelines for lipid management in people with DKD.^20^

Treatment is advised in people with diabetes (type 1 and 2) with:

• CKD G1–2 with albuminuria A2–3 and, if aged under 30 years, with an additional cardiovascular risk factor
• CKD stage 3–5 (treatment is advised regardless of additional risk factors).

Atorvastatin 20 mg is the starting point for treatment with up titration and addition of agents such as ezetimibe where targets are not met.

For simplicity, the targets of total cholesterol < 4 mmol/L, LDL cholesterol < 1.8 mmol/L and non-HDL cholesterol < 2.5 mmol/L were set.

## Conclusions

Early recognition and treatment of DKD is important to reduce cardiovascular disease risk and mortality. The five-finger rule is a simple way to ensure that people with DKD are managed appropriately. For people with type 2 diabetes, SGLT2i and finerenone have a significant impact on cardiorenal disease risk reduction. While these are not yet licensed in people with type 1 diabetes, ongoing studies will inform DKD management in the future. For all people with DKD, multifactorial intervention is effective and has a legacy effect.

## CRediT authorship contribution statement

**Sagen Zac-Varghese:** Writing – original draft.

## Declaration of competing interest

The authors declare that they have no known competing financial interests or personal relationships that could have appeared to influence the work reported in this paper.
